# A rapid bioluminescence assay for measuring myeloperoxidase activity in human plasma

**DOI:** 10.1038/ncomms7271

**Published:** 2015-02-10

**Authors:** Reece J. Goiffon, Sara C. Martinez, David Piwnica-Worms

**Affiliations:** 1 Mallinckrodt Institute of Radiology, Washington University School of Medicine, St Louis, Missouri 63110, USA; 2 Department of Cancer Systems Imaging, University of Texas MD Anderson Cancer Center, 1400 Pressler Street, Unit 1479, FCT16.6030, Houston, Texas 77030, USA

## Abstract

Myeloperoxidase (MPO) is a circulating cardiovascular disease (CVD) biomarker used to estimate clinical risk and patient prognosis. Current enzyme-linked immunosorbent assays (ELISA) for MPO concentration are costly and time-intensive. Here we report a novel bioluminescence assay, designated MPO activity on a polymer surface (MAPS), for measuring MPO activity in human plasma samples using the bioluminescent substrate L-012. The method delivers a result in under an hour and is resistant to confounding effects from endogenous MPO inhibitors. In a pilot clinical study, we compared MAPS and two clinical ELISAs using 72 plasma samples from cardiac catheterization patients. Results from parallel MAPS and ELISAs were concordant within 2±11 μg l^−1^ MPO with similar uncertainty and reproducibility. Results between parallel MAPS and ELISA were in better agreement than those between independent ELISAs. MAPS may provide an inexpensive and rapid assay for determining MPO activity in plasma samples from patients with CVD or potentially other immune and inflammatory disorders.

## Abstract (web summary)

Levels of the enzyme myeloperoxidase in the blood are considered a biomarker for the severity of cardiovascular disease. Here the authors report a rapid and inexpensive method for measuring myeloperoxidase activity in human plasma by luminescence, after adsorption of the enzyme to a polymer surface.

## Graphical abstract

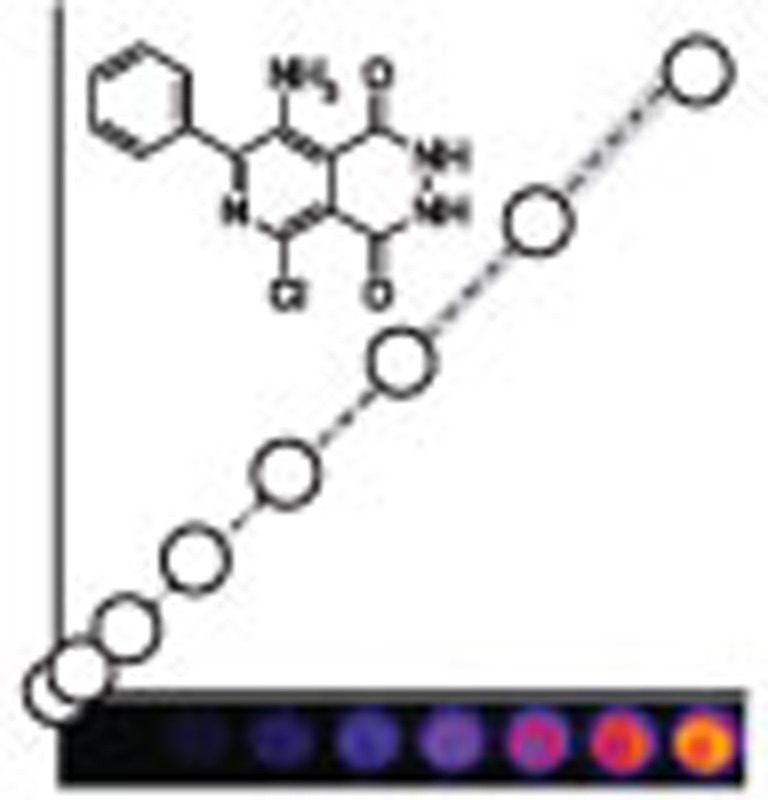

Atherosclerotic cardiovascular disease (CVD) is the leading cause of morbidity, mortality and health care costs in the developed world, a distinction that is projected to apply globally within the next decade^1^,^2^. Many metabolic and haemodynamic factors influence atherosclerosis progression, defined by arterial wall inflammation^3^. Atherosclerosis often first presents as a major adverse cardiovascular event (MACE), suggesting that identifying high-risk patients with subclinical disease before the first MACE is a vital prevention strategy^4^. Many proposed biomarkers for risk stratification target the inflammation underlying plaque development and instability^5^. The heme-containing antimicrobial enzyme myeloperoxidase (MPO) is one of these biomarkers.

MPO constitutes 5% of neutrophil dry weight and is concentrated in primary granules^6^. On neutrophil activation, these granules fuse to the phagosomal or cell membrane to oxidize biomolecules with hypochlorous acid produced by MPO^7^. Reactive oxygen species (ROS) generated by MPO can oxidize apolipoproteins, disrupt endothelial function and accumulate in the shoulder regions of plaques, suggesting a possible role in atherogenesis^8^,^9^.

Previous studies reviewed elsewhere have shown that circulating MPO levels correlate with measures of CVD severity and predict short- and long-term patient outcomes^10^,^11^. Plasma MPO concentration is usually measured by enzyme-linked immunosorbent assay (ELISA)^11^, which is costly, time-intensive and typically uses ROS generated by immunoconjugate horseradish peroxidase (HRP) instead of directly measuring MPO-derived ROS. Historically, attempts to measure MPO by its intrinsic activity show that this requires either immunologic purification or a chemically simple source^12^,^13^.

We have previously shown that MPO activity can be imaged directly *in vivo* with luminol, a chemiluminescent compound oxidized by hypochlorous acid^14^. L-012 is a luminol analogue that has also been used to measure ROS *in vivo* and *in vitro* with enhanced luminescence and sensitivity (Fig. 1a)^15^,^16^,^17^. Intracellular MPO concentrations at inflammation loci are high enough to oxidize bioluminescent probes for real-time, whole-animal imaging with charge-coupled device cameras, but circulating MPO is normally inhibited by proteins such as ceruloplasmin and antioxidants such as ascorbic acid^18^,^19^. Here we describe a new technique to assay MPO activity from whole plasma samples after eliminating inhibitors without requiring immunosorbent reagents or complex sample processing. This activity assay is simple, cost-effective and more sensitive than current ELISA techniques.

## Results

### MPO oxidizes L-012 by chlorination and bromination

**Figure 1:**
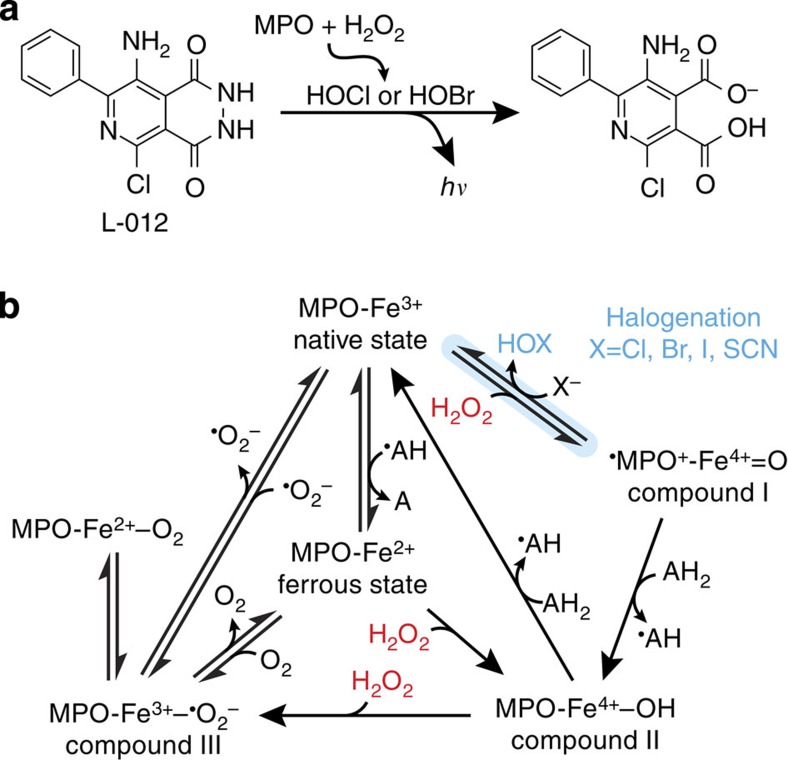
Biochemistry of MPO/L-012 bioluminescence. (**a**) Reaction of L-012 with ROS produced by MPO. Solution conditions can be optimized to effectively eliminate H_2_O_2_ chemiluminescence, rendering L-012 a bioluminescent reporter specific for MPO activity in living systems. (**b**) MPO has complex redox kinetics involving H_2_O_2_ and various electron carriers. Rapid halogenation (blue) is required to generate hypohalous acids. H_2_O_2_ (red) is both a halogenation substrate and inhibitor: excess H_2_O_2_ shifts MPO from halogenation with halide X^−^ and H_2_O_2_ into slower peroxidation cycles with electron donor AH_2_. Scheme adapted from Malle *et al*.^57^

**Figure 2:**
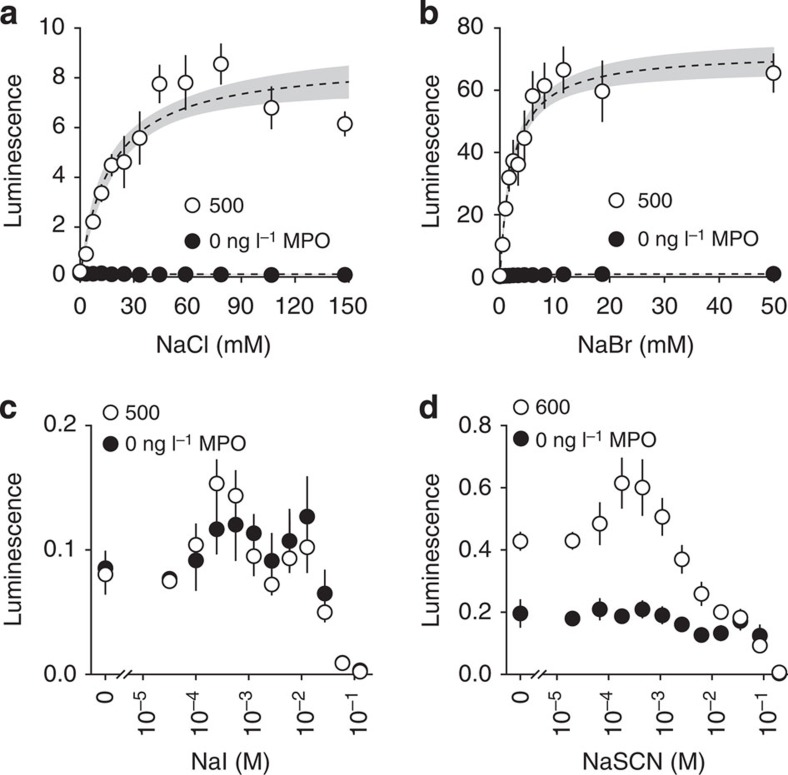
Halogenation substrates for MPO bioluminescence. (**a**) As suggested by the literature, MPO utilizes physiological concentrations of NaCl, *K*_m_=17 (10–23) mM, less effectively than (**b**) NaBr, *K*_m_=2.3 (1.7–2.9) mM. Although reported as even more efficient substrates for MPO, (**c**) I^−^ and (**d**) SCN^−^ do not produce similar MPO-mediated bioluminescence with L-012 under these reaction conditions. Representative data (*n*=4) are shown as mean±s.d.. Regression 95% confidence bands are shown in grey when applicable. MPO is labelled with final imaging concentration.

Full biochemical characterization of the MPO reaction under varied conditions was first pursued to optimize bioluminescence from the novel MPO activity assay. Although substrate interactions with MPO are far more complex than kinetics described by the Michaelis–Menten equation (Fig. 1b), NaCl and NaBr at relevant concentrations fit the model as classic substrates in terms of their effects on MPO-induced bioluminescence of L-012 (Fig. 2). In acidic citrate buffer, MPO bioluminescence plateaus near physiological extracellular NaCl concentrations with a *K*_m_=17 (10–23) mM. NaBr is a more efficient halogenation substrate with ~10-fold higher bioluminescence and affinity, *K*_m_=2.3 (1.7–2.9) mM. Contrary to literature reports, NaI and NaSCN did not generate ROS suitable for L-012 oxidation under these reaction conditions.

### Antioxidants in plasma inhibit MPO bioluminescence

**Figure 3:**
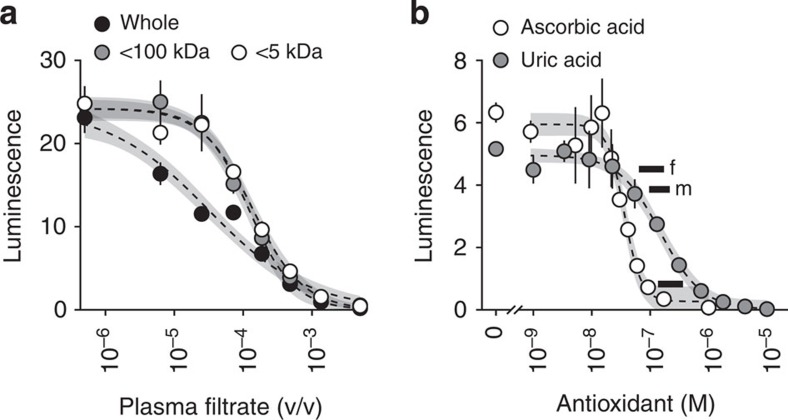
Plasma inhibits MPO-mediated bioluminescence. (**a**) Representative plasma filtrate titration curves in the presence of 500 ng l^−1^ MPO imaged with L-012 and H_2_O_2_. Whole plasma had a broad inhibitory concentration range with an IC_50_=28 (21–35) p.p.m. v/v. Removing plasma components >100 kDa narrowed the inhibitory concentration range, but only modestly increased the IC_50_ to 120 (110–140) p.p.m. Plasma components <5 kDa were not significantly different from <100 kDa in terms of inhibiting MPO bioluminescence (nonlinear regression *P*=0.21). (**b**) Endogenous antioxidant titration curves equivalent to plasma with 20 μg l^−1^ MPO imaged at a 0.4% v/v dilution. Ascorbic acid inhibition IC_50_=36 (31–41) nM, uric acid IC_50_=150 (120–180) nM in the presence of 80 ng l^−1^ MPO. The lower black bar spans the physiological plasma reference range of ascorbic acid adjusted by the same dilution factor; the upper black bars span the male and female uric acid reference ranges^20^. Representative data (*n*=4) are mean±s.d. Regression 95% confidence bands shown in grey.

Fresh plasma from healthy volunteers added to pure MPO inhibited bioluminescence with an IC_50_=28 (21–35) p.p.m. (Fig. 3a). Removing proteins >100 kDa via ultrafiltration narrowed the inhibitory concentration range and increased the IC_50_ to 120 (110–140) p.p.m. Further filtration to remove plasma components >5 kDa had no significant effect on IC_50_ (nonlinear regression *P*=0.21). Potent inhibition of MPO bioluminescence by endogenous plasma components <5 kDa suggests that MPO is predominantly inhibited by small-molecule antioxidants compared with the contribution from proteins such as ceruloplasmin in these reaction conditions.

Two potential endogenous inhibitors, ascorbic and uric acid, were titrated in the presence of MPO in solution at physiological ratios to directly quantify their antioxidant effects (Fig. 3b). Ascorbic acid inhibited MPO (80 ng l^−1^) with an IC_50_=36 (31–41) nM. These concentrations recapitulated pre-dilution human plasma containing 9 μM ascorbic acid (physiological reference range 34–91 μM)^20^ and 20 μg l^−1^ MPO. As pre-dilution ascorbic acid concentrations neared the high reference range cutoff, MPO signal was fully quenched. Uric acid also inhibited MPO, but showed a higher IC_50_=150 (120–180) nM. These data indicated that a direct plasma MPO activity assay would require elimination of antioxidants by dilution or selective removal. Unfortunately, dilution strategies were unsuccessful as plasma required dilution beyond the MPO detection threshold to effectively overcome antioxidant inhibition.

### MPO activity on a polymer surface (MAPS) assay

A strategy for assaying MPO from human plasma evolved from the biochemical analysis. Our initial findings indicated that MPO must first be isolated from native plasma inhibitors before its activity can be assayed with L-012 bioluminescence. This was accomplished by nonspecific adsorption of MPO onto a solid surface, which was then washed free of antioxidants. Pure MPO did not adsorb onto tissue culture-treated polystyrene alone, but strongly adsorbed when co-incubated with dilute human plasma (data not shown). Reconstituted bovine plasma (RBP) also allowed MPO adsorption and is preferable to human plasma in terms of availability and reduced biohazard potential.

**Figure 4:**
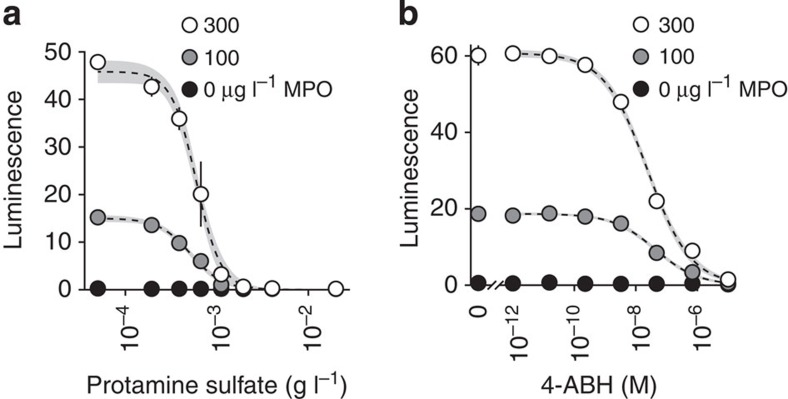
RBP facilitates MPO binding without contributing to bioluminescence. (**a**) RBP supplemented with MPO and titrated with protamine sulfate before loading onto a MAPS assay plate. MPO detection from supplemented RBP is inhibited with IC_50_=590 (560–620) μg l^−1^ protamine sulfate. Native RBP is not similarly affected by increasing concentrations of protamine sulfate and remains at baseline, demonstrating the lack of intrinsic MPO detectable by MAPS assay. All samples converge to native RBP at high concentrations of protamine sulfate. Regression confidence bands are shown in grey. (**b**) Titration curves of 4-aminobenzoic hydrazide (4-ABH) in MAPS imaging buffer on a plate loaded with RBP supplemented with human MPO. 4-ABH inhibited MAPS bioluminescence with an IC_50_=25 (22–28) nM. Native RBP was not inhibited. Data are plotted as mean±s.d. (*n*=4). MPO concentrations represent whole RBP, pre-dilution.

Native RBP contributed negligible signal to the MAPS assay as demonstrated by two methods. First, competitively blocking cationic protein adsorption, including MPO, eliminated any MAPS bioluminescence. For example, protamine sulfate completely inhibited detectable MAPS from RBP supplemented with human MPO with an IC_50_=590 (560–620) μg l^−1^ (Fig. 4a), but had no effect on native RBP signal, which remained at the baseline across the range of protamine concentrations as would be expected from plasma without intrinsic MPO activity. This finding was repeated with two different lots of RBP obtained independently. Second, 4-aminobenzoic hydrazide, a specific MPO antagonist^21^, titrated into the MAPS imaging buffer similarly inhibited bioluminescence from MPO-supplemented RBP with an IC_50_=25 (22–28) nM (Fig. 4b). 4-aminobenzoic hydrazide did not have this effect on native RBP. This also provided direct evidence that L-012 bioluminescence in the MAPS assay requires active MPO.

**Figure 5:**
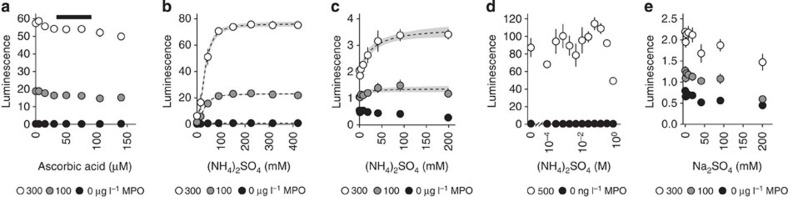
(NH_4_)_2_SO_4_ eliminates plasma protein inhibition of MPO bioluminescence. (**a**) RBP supplemented with purified human MPO and varying ascorbic acid concentrations prior to plate loading in a MAPS assay. Ascorbic acid is plotted at pre-dilution concentration in whole plasma. Black bar spans ascorbic acid reference range for human plasma^20^. (**b**) Titration curves of (NH_4_)_2_SO_4_ enhancing MAPS bioluminescence from human plasma and supplemented with MPO. Enhancement effect fits a sigmoidal ligand model with Hill coefficient=2.3 (2.0–2.5) and *K*_m_=34 (32–36) mM (NH_4_)_2_SO_4_ at the tested MPO concentrations. (**c**) Ammonium sulfate titration curves in the presence of H_2_O_2_, L-012 and varying MPO in plasma diluted to 0.4%. (NH_4_)_2_SO_4_ slightly increases MPO-dependent bioluminescence in human plasma, but not to the extent seen with MAPS. (**d**) Pure MPO in citrate imaging buffer is not enhanced by (NH_4_)_2_SO_4_ in the same manner as seen with MAPS. MPO concentrations represent post-dilution into the microtitre plate wells. (**e**) RBP supplemented with human MPO and imaged with varying Na_2_SO_4_, which has a mild inhibitory effect. Bioluminescence enhancement seen with (NH_4_)_2_SO_4_ shown in **b** requires NH_4_^+^ and is thus not an effect of ionic strength alone. Data are shown as mean±s.d. (*n*=4) with regression 95% confidence bands in grey. MPO is labelled as concentrations in whole plasma before dilution into loading buffer with the exception of final MPO concentrations in **d**.

Varying pre-assay plasma ascorbic acid concentrations had minimal effect on MAPS bioluminescence, demonstrating that the protocol mitigated the previously observed effects of small-molecule plasma MPO inhibitors (Fig. 5a). However, other specific MPO inhibitors, most notably ceruloplasmin, are known to adsorb onto treated polystyrene^22^. (NH_4_)_2_SO_4_ added to the MAPS imaging solution to disrupt non-covalent interactions between MPO and macromolecular inhibitors resulted in an ~10-fold increase in bioluminescence (Fig. 5b). This enhancing effect was modest with MPO assayed in solution format with diluted plasma containing native antioxidants, and altogether absent with MPO in pure buffer (Fig. 5c,d). Furthermore, Na_2_SO_4_ did not enhance MAPS bioluminescence at the same concentrations, confirming the requirement for NH_4_^+^ ions (Fig. 5e). Thus, the final MAPS assay protocol included (NH_4_)_2_SO_4_ in the imaging buffer to eliminate potential adverse effects of coadsorbed plasma inhibitors on MPO activity.

**Figure 6:**
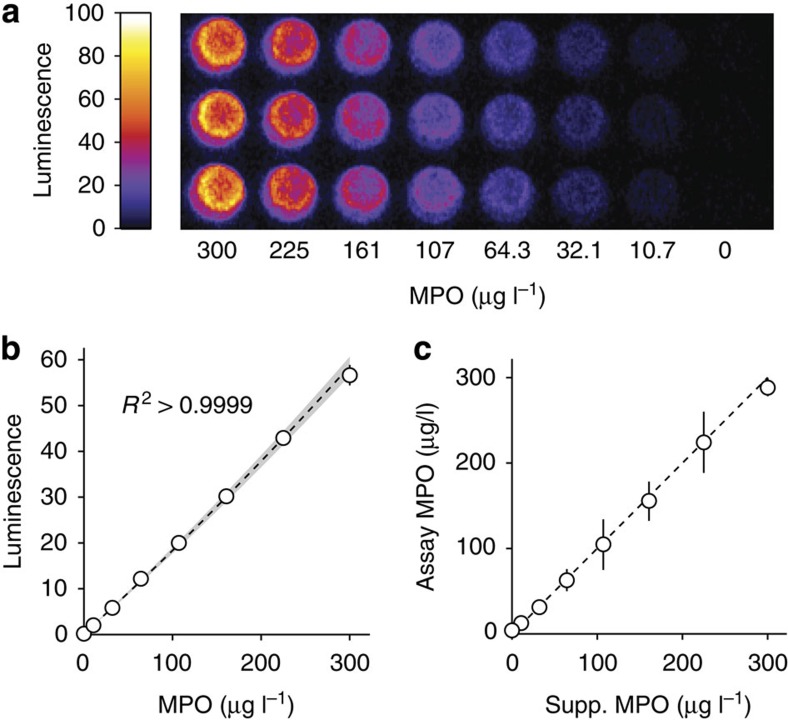
MAPS assay with RBP carrier. (**a**) Example standard solution image and (**b**) calibration curve constructed with purified human MPO diluted into RBP for MAPS assay. Data are plotted as mean±s.d. (*n*=3). Triplicate 95% prediction band shown in grey. (**c**) MAPS assay results using human plasma from healthy volunteers supplemented with purified human MPO to simulate samples covering the range of a commercial ELISA kit. Data are plotted as regressed value±95% confidence intervals. Dashed line represents 1:1 agreement between supplemented MPO and RBP-regressed MPO.

Using the MAPS technique, RBP supplemented with human MPO produced near-linear titration curves with high reproducibility (Fig. 6a,b). The MPO concentrations were chosen to cover the range suggested by commercial kit manufactureres and were skewed to improve resolution near literature prognostic thresholds (typically 30–70 μg l^−1^). MAPS was precise by weighted least squares (WLS) regression analysis (*R*^2^>0.999) and used plasma diluted 10-fold beyond requirements for ELISA. Furthermore, to simulate clinical samples, human plasma from healthy volunteers was then supplemented with incremental MPO concentrations and calibrated against MPO standards in RBP (Fig. 6c). Results from this simulated MAPS assay consistently agreed with the known MPO values within 95% confidence limits.

### Clinical study population characteristics

We next applied the MAPS assay in a pilot clinical trial of human samples. The clinical characteristics of the patient cohort as they presented prior to elective cardiac catheterization are shown in Table 1. We recruited patients with diverse demographic and clinical profiles. As expected from the selection criteria, average plasma MPO concentration in this cohort was slightly lower than literature reports on higher-risk patients, such as those presenting with acute coronary syndrome (ACS). Of note, 17 patients had received i.v. heparin prior to sampling. We previously observed that plasma collected from heparinized vacuum tubes did not have strong MAPS bioluminescence, indicating that heparin at high concentrations may block MPO adsorption by a mechanism similar to protamine sulfate. Despite this possibility, samples from patients treated with heparin had detectable MAPS bioluminescence and were included in this analysis.

### Comparative measurement of human plasma MPO

Seventy-two plasma samples were analyzed by MAPS and an ELISA kit protocol modified to minimize procedural variance and allow direct, quantitative comparison (that is, assays ‘in parallel’). Blocks of 24 plasma samples and 8 MPO standard curve solutions spanning the concentration range recommended by the kit manufacturer were analyzed in triplicate. In addition, 67 plasma samples with adequate volume were also analyzed independently by the Cleveland HeartLab (CHL), LLC (Cleveland, OH, USA), which offers Food and Drug Administration (FDA)-approved, ELISA-based clinical MPO assay services. Differences in protocol and reported units limit quantitative comparison between the CHL results and those from either parallel assay.

**Figure 7:**
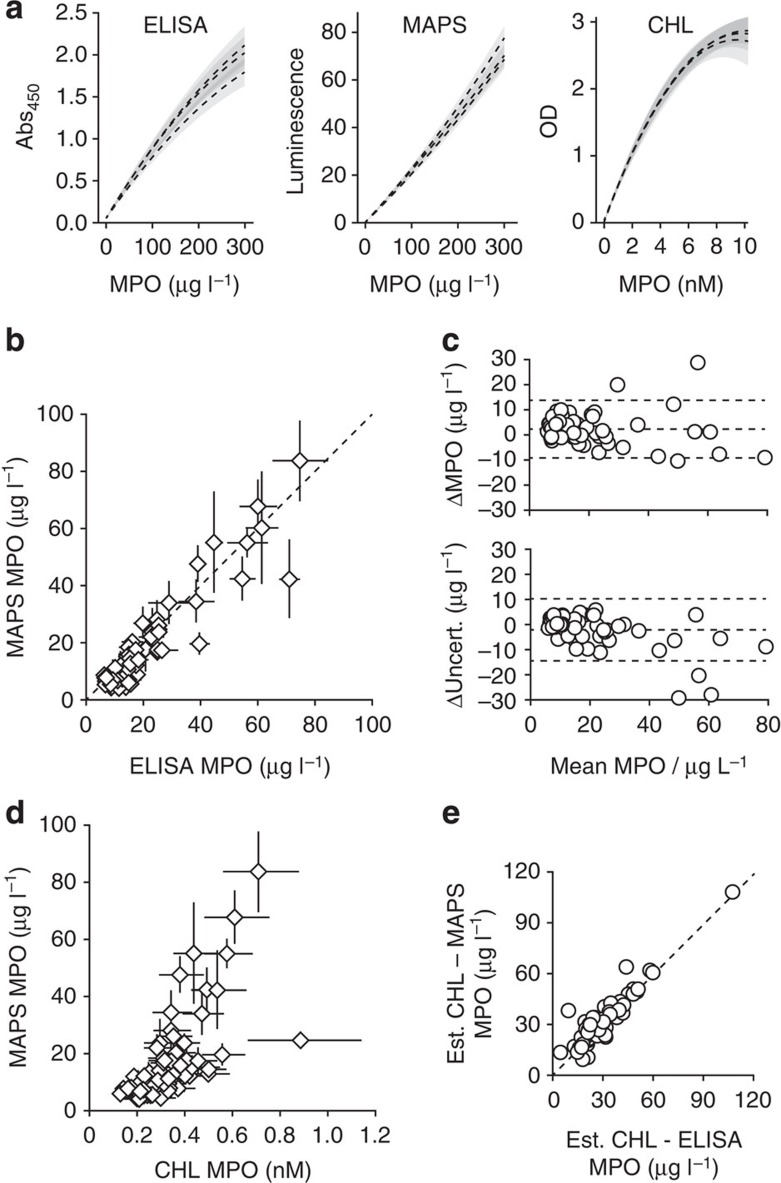
MAPS and ELISA with clinical plasma samples. (**a**) Three calibration curves with triplicate prediction bands. The mean limit of detection for MAPS was significantly lower than for ELISA, 6.0 (5.3–6.6) and 7.4 (6.7–8.1) μg l^−1^, respectively (generalized linear regression *P*=0.002). (**b**) Regressed plasma MPO from 72 clinical plasma samples. Error bars show combined uncertainty from calibration curves and inter-replicate variance by WLS regression. The assays are not significantly different from perfect agreement (dashed line) by either unweighted (*P*=0.46) or uncertainty-weighted (*P*=0.61) matched pair linear regression. (**c**) Discrepancy (ELISA–MAPS) of both MPO result and uncertainty. Dashed lines are means and 95% coverage limits. The assays agreed within 2±11 μg l^−1^ MPO and varied in uncertainty by −2±12 μg l^−1^ MPO. (**d**) Results of the MAPS and CHL assays from 67 clinical plasma samples. Standardization unit disagreement limits analysis to correlation calculated, respectively, as Pearson *r*=0.90 and 0.86 when weighted and unweighted by combined uncertainty. (**e**) CHL results differ similarly from MAPS and protocol-matched ELISA. CHL-ELISA and CHL-MAPS disagreement is not significantly different across the cohort (generalized linear regression *P*=0.20). CHL data were converted to microgram per litre by an estimate for MPO molecular weight for direct comparison.

The WLS calibration curve parameters consistently differed between the two methods: both ELISA curves were moderately concave owing to the saturable nature of absorbance measurements, while MAPS curves were linear or marginally convex (Fig. 7a). Concavity reduces overall assay resolution, as evident by the statistically significant difference in the computed limits of detection: 6.0 (5.3–6.6) μg l^−1^ for MAPS and 7.4 (6.7–8.1) μg l^−1^ for the parallel ELISA (generalized linear regression *P*=0.002).

MPO determined by the two parallel methods was highly correlated (Pearson *r*=0.97, Fig. 7b and Table 2). The global disagreement between the two assays was not significant (matched pair generalized linear regression *P*=0.46). Disagreement that did exist was largely due to the combination of both data set uncertainties. To adjust for this, we weighted each plasma sample by the inverse product of assay uncertainty, resulting in even less deviation from agreement (weighted generalized linear regression *P*=0.61).

Although parallel MAPS and ELISA agreed over this entire cohort, individual plasma measurements deviated with 95% limits of agreement of 2±11 μg l^−1^ MPO (Fig. 7c). Uncertainty, which accounts for variance in both the calibration curve and individual plasma samples, was also consistently distributed between the two assays: ELISA uncertainty with 95% coverage was −2±12 μg l^−1^ compared with that of MAPS.

MAPS and the CHL assay were also well-correlated (uncertainty-weighted and unweighted correlation *r*=0.90 and 0.86, respectively, Fig. 7d and Table 2). Although precise quantification of the disagreement is limited by the difference in calibration standards, the agreement of both parallel assays with the CHL results can be compared by using an approximate MPO molecular weight of 150,000 kDa. The overall disagreement of both the MAPS assay and parallel ELISA protocol with the CHL results were strongly correlated (*r*=0.99) and did not significantly vary with MPO as paired data across patients (generalized linear regression *P*=0.20, Fig. 7e). Of note, this demonstrates that varying protocol details for mechanistically similar assays, in this case two ELISAs performed at different sites, is more detrimental to reproducibility than is the comparatively large operational difference between how the measured quantity is produced in MAPS versus ELISA.

### MAPS assay reproducibility

**Figure 8:**
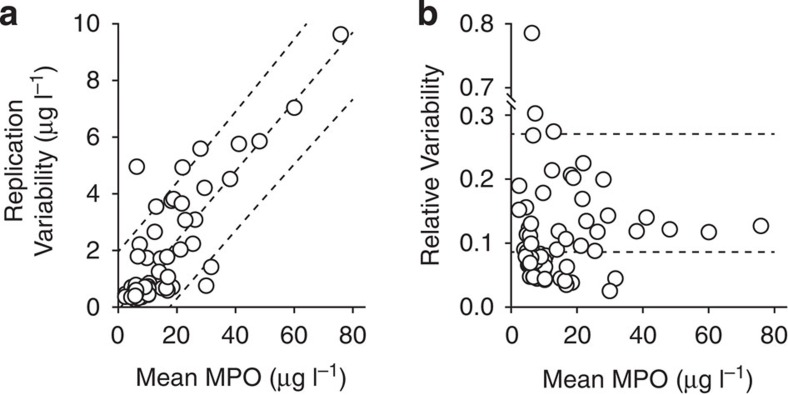
MAPS reproducibility. MAPS assays independently replicated four times on 72 clinical plasma samples. Dashed lines show median and one-sided 95th percentile values. Computation details are provided in online methods. (**a**) Absolute replication variability quantified by accounting for both intra- and inter-replicate uncertainty. Median variability was 0.67 μg l^−1^ MPO, and 95% of samples were reproducible within 5.8 μg l^−1^ MPO. Variability was scaled with mean MPO by a factor of 0.12 (0.11–0.13). Dashed lines show mean and two-sided 95% coverage variability estimates. (**b**) Relative replication variability by normalization to variance-weighted replicate mean MPO. Median coefficient of variability was 8.6% with an upper 95th percentile threshold of 27%. Dashed lines show median and one-sided 95th percentile estimate.

We performed additional, independent replications of the MAPS assay with all 72 patient samples on 3 separate days. By combining the inter- and intra-assay replication variances from these and the initial replicates, we were able to calculate absolute replication variability for each sample (Fig. 8a). The median and 95th percentile replication variability scores were 0.67 μg l^−1^ MPO and 5.8 μg l^−1^ MPO, respectively. Variability was proportional to mean MPO by a ratio of 0.12 (0.11–0.13). By normalizing these values to the replicate means, we computed a more robust version of the coefficient of variation (Fig. 8b). This also makes the difference between two sample groups—those with homoscedastic and heteroscedastic variability—more apparent. Even when including the heteroscedastic samples, the median coefficient of variability was 8.6% with 95th percentile coverage by 27%.

## Discussion

MPO is an inflammation biomarker that has been shown to be independently associated with acute and chronic risk of MACEs in patients with established CVD, presenting with ACS, or undergoing diagnostic cardiac catheterization^10^,^11^,^23^. Serial measurements are predictive of acute progression from ACS to a MACE in patients with negative troponin I levels^24^. It has also been shown to predict CVD development in otherwise healthy individuals^25^. In this study, we show that human plasma MPO can be assayed by a conceptually new method, MAPS, as an alternative to ELISA. The MAPS assay method is advantageous to ELISA in terms of cost, time, complexity and sample consumption. Increasing the clinical stability and accessibility of an MPO assay allows for investigation of CVD risk stratification, outcomes and management.

The measured quantity of our MAPS assay fundamentally differs from that of ELISA: instead of using the activity of immunoconjugated HRP to measure the mass concentration of MPO, MAPS directly measures MPO enzyme activity. Both types of measurement are hypothetically subject to confounding factors. The MAPS assay assumes equivalent enzyme activity compared with the reference standard and could underestimate MPO concentration should a patient have hypomorphic MPO. Both our observations and those in the literature suggest that the assumption of constant activity is reasonable^26^.

Unintuitively, ELISA has potential for the same problem: MPO and HRP can both catalyze the reaction responsible for the change in 450 nm absorbance. In addition, ELISA also requires the assumption of antigenic homogeneity. Thus ΔAbs_450_ is not a linear function of MPO concentration, but rather a combination of MPO activity, HRP activity and the combined affinity of two different antibodies for sample MPO. Although this study did not identify any patients with highly discrepant results between the methods, we cannot assume that mutations and other confounding factors would have no effect in a much larger population.

As a general technique, ELISA is considered to be robust and reliable. Disagreement in ELISA results can come from external sources (such as operator technique, sample handling or random variation) or intrinsic sources (such as antibody affinities, epitope frequency or nonspecific effects of reagents and buffers on the analyte). Both the CHL and bench-top kit ELISAs are well-validated and thus the intrinsic sources of discrepancy are minimal compared with the conceptual intrinsic differences between ELISA and MAPS. From this, we initially hypothesized that the two ELISAs would correlate more with each other than either would with MAPS. We tested this hypothesis by minimizing the extrinsic differences between an ELISA and the MAPS assay by performing them in parallel. We instead found that the CHL-ELISA, which was automated and performed by the CHL unmodified internal protocol, agreed less with our ELISA than did our parallel-protocol MAPS assay. The overall distribution of data shows that the CHL assay has an elevated baseline and greater relative uncertainty in the low-end of MPO concentrations compared with MAPS and the parallel ELISA. In patient samples exceeding ~30 μg l^−1^, the CHL and MAPS data agree linearly within uncertainty limits. Only one patient was an obvious outlier, but this sample produced highly variable data from the CHL assay and thus cannot be confirmed as a true outlier with confidence.

Some possible sources of this variation include the time passed between the parallel assays and the CHL measurements, effects of sample shipment to the control lab, standard curve concentration intervals and execution by automated fluid handlers versus hand pipetting. That these unavoidable extrinsic sources of variation had a larger effect than the conceptual difference between the parallel ELISA and the MAPS assay demonstrates how suitable MAPS is as a substitution for ELISA. Our data suggest that MPO activity measured with MAPS and concentration as measured by ELISA are sufficiently correlated in the range of plasma MPO expected from clinical samples.

Although similar to ELISA in terms of results and reproducibility, we propose that the MAPS assay has many practical advantages. Most obvious is the difference in cost: MAPS does not require antibodies or assay-specific labware. We estimate the cost per-patient as <5% than that of ELISA. In addition, ELISA requires numerous reagent exchanges and incubations spanning many hours. The MAPS assay required only one incubation for which we arbitrarily chose 30 min for consistency. Further pilot data suggested that this can be shortened to 10 min with comparable MPO resolution. MAPS also appears to be more linear than ELISA, in part because luminescence is less saturable than absorbance. Higher sensitivity also allows MAPS to be measured from plasma samples diluted 10-fold from that required by standard ELISA, further extending the dynamic range in addition to requiring far less sample material.

There may be limitations to the MAPS method. At this time, the plasma components required for coadsorption are unknown. We unsuccessfully tested many pure carrier proteins, but only whole RBP gave reproducible, linear responses to MPO. We also found that heparinized phlebotomy tubes reduced MPO adsorption. Not only does this raise concern over i.v. heparin potentially interfering with the assay, but also suggests that the effects of other plasma components need to be further investigated. There was no statistically significant trend among the samples from patients treated with i.v. heparin for lower MAPS results compared with ELISA, but our study was neither designed nor powered to investigate this.

Although most literature investigating the clinical usefulness of MPO shows positive results, there are also examples of negative studies. When considering conflicting results, it is important to note the effect of protocol on MPO measurements. Previous work shows that MPO can be influenced by processing time, temperature and anticoagulants^27^,^28^,^29^. In studies of patients presenting with chest pain or proven myocardial infarction, systemic administration of heparins is another potentially confounding factor^30^,^31^. Studies with weak or negative results often lack detailed descriptions of protocols used to collect samples^32^,^33^,^34^, used heparin-treated tubes^35^ or measured MPO from serum^36^. In a notable negative study with MPO measured in EDTA-treated samples collected from heparin-naïve patients^37^, MPO concentration was log-transformed due to its skewed distribution; not only is such a transformation unnecessary, but it can obfuscate the true underlying relationships between covariates and outcomes^38^. These issues in methodology and analysis underscore the need for more investigation of MPO and its clinical utility. Our pilot study demonstrates the fundamental feasibility of the MAPS assay in a clinical setting, which we hope encourages such research in the near future.

Although we used an advanced imaging system in developing this assay, MAPS can be measured with common laboratory equipment. We successfully performed MAPS assays using a standard digital camera intended to photograph gel electrophoresis bands. Microtiter plate luminometers also open the assay to high-throughput automation. Because this assay only requires a hydrophilic polystyrene surface, the MAPS assay is amenable to a dipstick platform similar to blood glucose meters.

We have developed the assay in the context of CVD, but there are other important clinical scenarios that could benefit from rapid MPO measurement to assess inflammation and immune response. A growing number of studies have suggested prognostic value of MPO in the setting of cancer. MPO concentration was shown to differentiate patients with hepatocellular carcinoma from those with chronic hepatitis or severe cirrhosis in the context of hepatitis C^39^. MPO has been shown to be elevated in plasma from patients with glioblastoma multiforme and numerous gynaecological cancers; bile MPO has also been used to differentiate benign choledochal strictures from cholangiocarcinoma^40^,^41^,^42^. In addition, research suggests that neutrophils laden with MPO infiltrating colorectal cancer may have a role in the anti-tumor immune response^43^,^44^. Cardiotoxicity from chemotherapy is also predicted by MPO levels in patients with breast cancer^45^. The MAPS assay has potential uses outside of oncology as well. Serial MPO levels made possible by a rapid, cost-effective assay could improve detection of post-transplant graft rejection as MPO rises in patients rejecting heart and liver allografts^46^,^47^. If developed onto a more rapid or portable platform, MPO activity may also be useful in diagnosing early wound infections, urinary tract infections and advanced progression of septic shock in the acute critical care setting^48^,^49^,^50^.

In conclusion, we have developed a facile MPO activity assay applicable to clinical plasma samples as an alternative to ELISA. The MAPS assay has many practical advantages including a >10-fold reduction in cost, sample material consumption and labour requirement. Importantly, we found the MAPS assay to be comparable to ELISA in results, uncertainty and reproducibility in a test with 72 cardiac catheterization patient samples.

## Methods

### Materials

L-012 (product no. 120-04891) was supplied by Wako Chemicals USA (Richmond, VA, USA). Human MPO ELISA kits (product no. ADI-900-115) were supplied by Enzo Life Sciences (Farmingdale, NY, USA). Purified human MPO (product no. 475911) was supplied by EMD Millipore/Calbiochem (Darmstadt, Germany). Bovine plasma (product no. P4639) and all other reagents were supplied by Sigma-Aldrich (St Louis, MO, USA). Bioluminescence readings were obtained with the Stanford Photonics ONYX/M imaging system (Palo Alto, CA, USA). All bioluminescence experiments were performed in Corning black-walled, 96-well microtitre plates made from tissue culture-treated polystyrene (product no. 3603).

### Human plasma samples

*Plasma for assay development*. Blood was drawn from the cephalic vein of volunteers undergoing elective coronary catheterization (37 men and 38 women, age range=30–89 with mean=63, see Table 1 for other characteristics) into K_2_EDTA vacuum tubes for immediate preparation. Plasma was isolated by two-stage centrifugation at 4 °C: 1,000*g* in the collection tubes for 15 min followed by 14,000*g* in microcentrifuge tubes for 10 min. *Clinical plasma samples*: cardiac patients undergoing elective catheterization were asked to allow collection of ~10 ml of blood by a physician after successful and uncomplicated arterial cannulation. Blood was transported on ice in K_2_EDTA vacuum tubes for plasma collection via centrifugation in the same manner described above. Plasma was stored at −80 °C and thawed on ice before use. Samples sent to the CHL had no attached personal information and were identified only by randomly generated demographics. The Institutional Review Board at Washington University in St Louis approved these study protocols. All participants provided written and informed consent at enrolment.

### Solutions

Stock solutions listed below were prepared from concentrated individual components and sterilized by microfiltration into 50 ml conical tubes. Additional solution components are described by experiment.

Cit6: 3.1 mM citric acid, 21.9 mM trisodium citrate

PS6.5: 130 mM NaCl, 6.28 mM Na_2_HPO_4_, 18.7 mM NaH_2_PO_4_

MPO dilution buffer: buffer PS6.5 with 200 p.p.m. v/v Tween20

MAPS loading buffer: buffer PS6.5 with 50 p.p.m. v/v Tween20

MAPS imaging solution: buffer Cit6 with 20 mM NaBr, 200 mM (NH_4_)_2_SO_4_, 100 p.p.m. v/v Tween20, 50 μM L-012 and 100 μM hydrogen peroxide (H_2_O_2_)

### Bioluminescence imaging

All images were acquired with the ONYX/M imaging system with a XR/MEGA-10Z camera at 55 frames per second with no optical filter. Images were integrated for 10 s to prevent camera saturation and compiled into 1 min exposures for quantification with the Fiji distribution of ImageJ analysis software^51^. Optical vignetting was mathematically corrected by the standard irradiance equation calibrated to the imaging system prior to quantification^52^. All bioluminescence data are presented in dimensionless intensity units and are uniformly scaled throughout to allow direct comparison between experiments.

### MAPS assay with RBP

All volumes were added by reverse pipetting to minimize variance from solution viscosity. Purified human MPO standards were prepared at full concentrations via serial dilution into MPO dilution buffer. One 1.2 ml capacity polypropylene cluster tube for every sample was filled with 500 μl MAPS loading buffer, each of which then received 2 μl of a plasma sample. Assay standards were similarly prepared by filling cluster tubes with 500 μl MAPS loading buffer, adding 2 μl of a standard MPO solution and finally adding 2 μl whole RBP. All tubes were capped tightly and thoroughly mixed on a vortex mixer. Samples were then loaded into microtiter plate wells as 100 μl volumes if done in triplicate (for example, Figs 6b, 7 and 8) or as 75 μl if done in quadruplicate (for example, Fig. 5a,b) based on pipette constraints. The microtiter plate was covered and shaken for 30 min at 1,000 r.p.m.. Each well was vacuum-aspirated dry without contacting the well surface and washed three times with MAPS loading buffer: twice with 150 μl then once with 300 μl. Fresh MAPS imaging solution was prepared with L-012 and H_2_O_2_ was added immediately before use. Each well received 75 μl imaging solution and the plate was briefly shaken up to 1,000 r.p.m. and imaged immediately. Data were quantified at the 5-min time-point.

### L-012 and MPO halogenation

All solutions were made in buffer Cit6. Reactions were set up adding 75 μl buffered halide to 50 μl of either MPO or buffer in microtiter plate wells. Thiocyanate, known as a ‘pseudohalide’ due to its chemical similarity to true halides, was analyzed in the same manner. Reactions were initiated by adding 150 μl buffered imaging solution with final concentrations of 100 μM H_2_O_2_ and 30 μM L-012. NaCl and NaBr titration curves were fit to the Michaelis–Menten equation.

### MPO inhibition by plasma

Fresh plasma from a healthy donor was diluted to 0.5% in buffer Cit6 with 10 mM NaBr. Dilutions were filtered by centrifugation in Corning Spin-X UF concentrators with 100 and 5 kDa cutoffs (product numbers 431486 and 431482) spun for 60 min at 4,000 *g* and 4 °C. Diluted whole plasma was prepared the same way and stored at 4 °C for the duration of centrifugation to control for temperature effects. Serial dilutions of each filtrate were made with final concentrations 10 mM NaBr, 50 μM L-012 and 500 ng l^−1^ MPO in the same buffer. Bioluminescence was initiated by adding 75 μl of these solutions to a plate arrayed with 10 μl buffered H_2_O_2_, final concentration 50 μM. Plates were shaken briefly up to 1,000 r.p.m. and imaged. Inhibition curves were fit to the equation


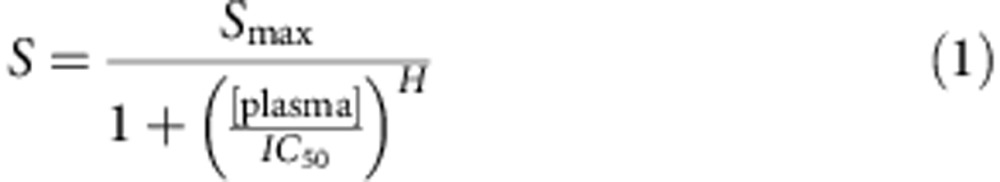


where *S* is bioluminescence signal with asymptote *S*_max_, IC_50_ is the concentration of plasma at which 
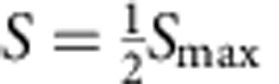
, and *H* is the Hill coefficient.

### MPO inhibition by plasma antioxidants

Serial dilutions of ascorbic acid were made in buffer Cit6 with final concentrations 30 μM L-012, 80 ng l^−1^ MPO and 200 p.p.m. Tween20. Bioluminescence was initiated by adding 75 μl of each antioxidant titration solution to 75 μl buffered H_2_O_2_, final concentration 100 μM. These concentrations of MPO and antioxidants were chosen to represent plasma from a healthy individual with 20 μg l^−1^ MPO diluted to 0.4% (ref. 20). Inhibition was modelled with a modified version of equation (1) that has a term for a non-zero asymptotic minimum:


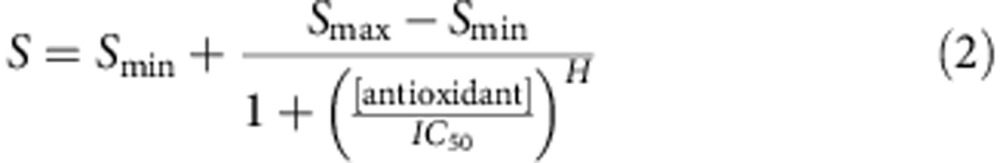


### MPO ELISA

ELISAs on clinical samples were performed in our laboratory with slight modification to the manufacturer’s suggested protocol to minimize systematic bias between ELISA and MAPS results. The assay volumes and liquid handling techniques were matched as closely as possible. The standard curve dilution series was matched to that used in MAPS assays. Plasma and MPO standard curve samples were loaded and analyzed in triplicate. Plasma samples of adequate volume to meet the requirements stated by the CHL were shipped overnight on ice for independent analysis. The CHL assay was performed as independent triplicates by CLIA-certified CHL, LLC (Cleveland, OH, USA) laboratory staff and automated fluid handlers. Raw instrument output was obtained from the CHL for analysis.

### Statistical analysis

All statistical analyses were performed with IBM SPSS Statistics 21 (IBM Corp., Armonk, NY, USA). Presented data are representative of three independent replications unless otherwise noted. Regression and measurement means are presented with 95% confidence intervals in parentheses throughout the text. Nonlinear regression confidence bands were calculated using a modified delta method with values of the gradient function estimated by the SPSS nonlinear regression algorithm^53^.

Calibration confidence and prediction bands (see Figs 6b and 7a) for ELISA and MAPS were computed using a WLS regression algorithm adapted from previous literature^54^,^55^. Calibration solutions were weighted by the inverse of the variance of the intra-experiment replicates. Variances for the continuous calibration bands were modelled as a function of measurement mean in the calibration solutions, 
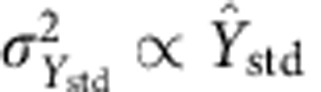
. Linear or quadratic calibration models were chosen by the algorithm using the *F*-ratio test. Plotted prediction bands were constructed using the assumption of triplicate measurements from a given unknown sample. MAPS and ELISA were analyzed identically from experiments run with triplicate wells. MAPS-independent replications were not combined in comparison against the single ELISA to keep from artificially reducing MAPS result uncertainty. ΔMPO was calculated as MPO_ELISA_—MPO_MAPS_ and Δuncertainty is the analogous difference in 95% confidence interval magnitude.

MAPS reproducibility was calculated with a method adapted from published techniques^56^. Each plasma sample MAPS replicate *i* (out of *k* total) was first weighted by the inverse of its estimate variance as determined by the WLS confidence interval.


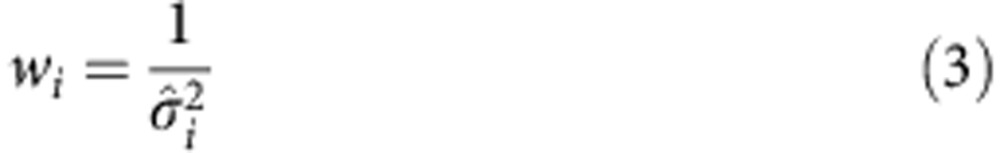


A weighted mean 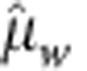 of the regressed MPO values 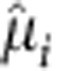 was then calculated for each plasma sample


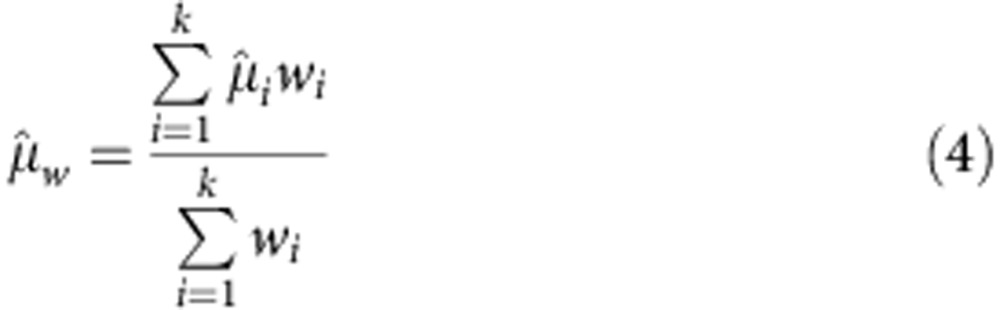


From these weighted means, a term 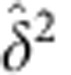 describing the variance of replication was computed


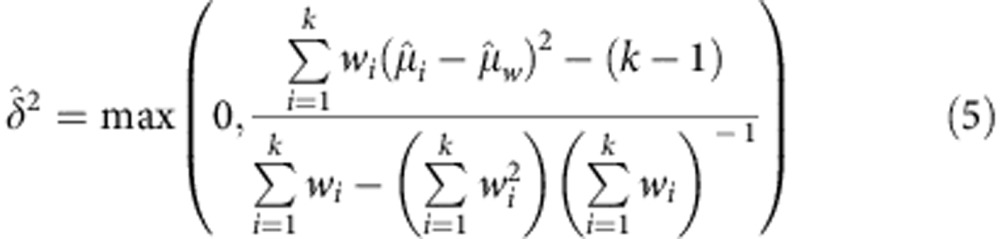


This term was combined with individual replicate uncertainty to compute a new weight and from this a weighted mean accounting for intra- and inter-assay variance.


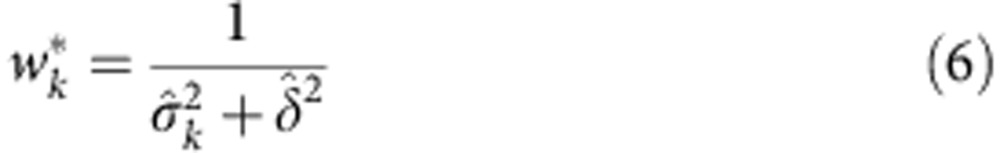



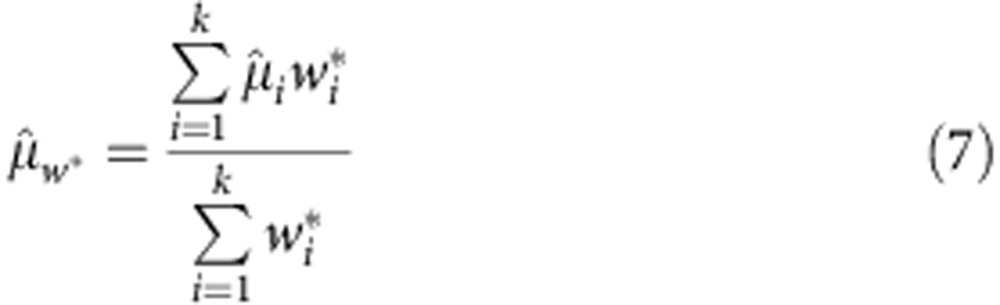


The absolute and relative replicate variability scores shown in Fig. 8 are, respectively:


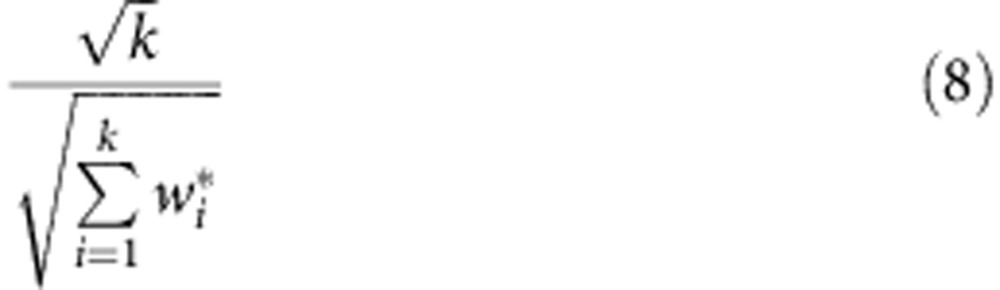


and


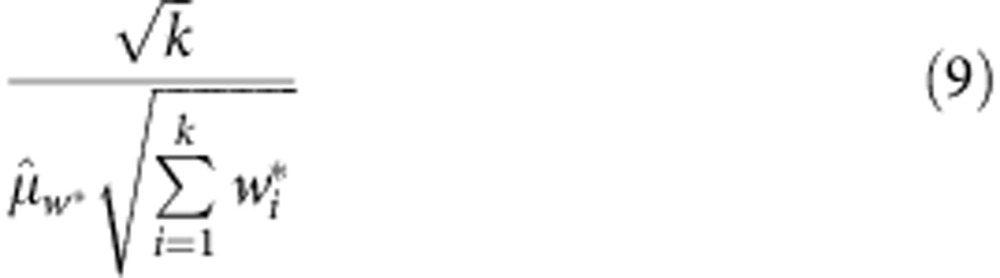


## Author contributions

All authors contributed extensively to direction, discussion and analysis throughout project. R.J.G. designed and performed the experiments, analyzed the data and wrote the manuscript. S.C.M. designed the clinical trial, built and maintained the sample library, and edited the manuscript. D.P-W. guided the experiments, supervised the analysis and edited the manuscript.

## Additional information

**How to cite this article:** Goiffon, R. J. *et al*. A rapid bioluminescence assay for measuring myeloperoxidase activity in human plasma. *Nat. Commun*. 6:6271 doi: 10.1038/ncomms7271 (2015).

## Figures and Tables

**Table 1:** Clinical data from plasma donors.

*N*	72*
Age (years)	63±14
Female:Male	35:37
Eur:Afr:Asn:Hisp	54:15:2:1
BMI (kg m^−2^)	29±7
Family CVD history	34 (6)
Current smoker	39 (1)
Hypertension	58
Diabetes mellitus	23
Known CAD	36
Prior PCI	21
Prior CABG	8
Total cholesterol (mg dl^−1^)	172±48
LDL (mg dl^−1^)	98±37
HDL (mg dl^−1^)	46±22
Aspirin	49
β-blockers	41 (1)
Statins	39
P2Y_12_ inhibitors	21
Heparin	17
p-ELISA MPO (μg l^−1^)	20±16
MAPS MPO (μg l^−1^)	14±13
CHL MPO (pM)	330±140

Afr, African; Asn, Asian; BMI, body mass index; CABG, coronary artery bypass graft; CAD, coronary artery disease; CVD, cardiovascular disease; ELISA, enzyme-linked immunosorbent assay; Eur, European; FDA, Food and Drug Administration; Hisp, Hispanic; MAPS, MPO activity on a polymer surface; MPO, myeloperoxidase; PCI, percutaneous coronary intervention.

Mean±s.d. are given when applicable. Parentheses indicate absent patient data. p-ELISA refers to the parallel-protocol ELISAs modified to approximate the MAPS protocol as closely as possible and eliminate systematic error. CHL is the FDA-approved MPO assay performed independently by the Cleveland HeartLab.

^*^CHL *N*=67 due to limited sample volume.

**Table 2:** Pearson correlations between clinical MPO assay results.

	**MAPS**	**p-ELISA**	**CHL**
MAPS	—	0.969	0.860
p-ELISA	0.960	—	0.893
CHL	0.895	0.911	—

ELISA, enzyme-linked immunosorbent assay; MAPS, MPO activity on a polymer surface; MPO, myeloperoxidase; p-ELISA, parallel-protocol ELISA.

Upper right values are unweighted, pairwise correlations. Lower left values were obtained by weighting patient samples by the inverse product of uncertainties from each correlated pair of assays.
